# Supplementary material for: Minerals and Antioxidant Micronutrients Levels and Clinical Outcome in Older Patients Hospitalized for COVID-19 during the First Wave of the Pandemic
Source: Nutrients. 2023 Mar 21;15(6):1516. doi: 10.3390/nu15061516 (PMC10056386; doi:10.3390/nu15061516)
Supplement: Supplementary file 1 [file nutrients-15-01516-s001.zip › nutrients-2246093-supplementary.pdf]

Supplementary Table S1. Logistic regression analysis of association between the 41 severe COVID-19 and serum antioxidant micronutrients levels

Severe COVID-19 (Yes/No) and Zinc								
	Univariate				Multivariate			
Variable	Crude OR	95% CI		p-value	adjusted OR	95% CI		p-value
Age > 75 years	0.595	0.258	1.375	0.2246	0.197	0.034	1.133	0.0687
Male sex	1.510	0.665	3.428	0.3242	0.586	0.151	2.279	0.4409
CRP (mg/L)*	1.005	1.000	1.011	0.0614	0.992	0.981	1.003	0.1605
Vit D supp	0.583	0.245	1.387	0.2225	0.639	0.167	2.446	0.5133
Albumin (g/L) **	0.741	0.646	0.850	<0.0001	0.681	0.564	0.823	**<.0001**
BMI ⩾ 35 (kg/m²)	2.765	0.646	11.838	0.1706	24.313	1.083	545.751	**0.0444**
Zinc (mg/L) #	1.411	1.057	1.884	**0.0196**	2.133	1.139	3.993	**0.0179**

Severe COVID-19 (Yes/No) and Selenium								
	Univariate				Multivariate			
Variable	Crude OR	95% CI		p-value	adjusted OR	95% CI		p-value
Age > 75 years	0.595	0.258	1.375	0.2246	0.282	0.056	1.429	0.1262
Male sex	1.510	0.665	3.428	0.3242	0.461	0.113	1.881	0.2803
CRP (mg/L)*	1.005	1.000	1.011	0.0614	0.998	0.989	1.008	0.7568
Vit D supp	0.583	0.245	1.387	0.2225	0.622	0.151	2.562	0.5109
Albumin (g/L) **	0.741	0.646	0.850	**<0.0001**	0.730	0.608	0.876	**0.0007**
BMI ⩾ 35 (kg/m²)	2.765	0.646	11.838	0.1706	2.106	0.015	286.683	0.7664
Selenium (µg/L) ###	1.120	0.890	1.410	0.3345	1.157	0.752	1.780	0.5065

Severe COVID-19 (Yes/No) and Copper								
	Univariate				Multivariate			
Variable	Crude OR	95% CI		p-value	adjusted OR	95% CI		p-value
Age > 75 years	0.595	0.258	1.375	0.2246	0.587	0.156	2.206	0.4305
Male sex	1.510	0.665	3.428	0.3242	0.503	0.140	1.809	0.2925

CRP (mg/L)*	1.005	1.000	1.011	0.0614	0.998	0.989	1.008	0.7383
Vit D supp	0.583	0.245	1.387	0.2225	0.517	0.139	1.914	0.3230
Albumin (g/L) **	0.741	0.646	0.850	**<0.0001**	0.691	0.566	0.844	**0.0003**
BMI ⩾ 35 (kg/m²)	2.765	0.646	11.838	0.1706	11.622	0.829	162.844	0.0686
Copper (mg/L) #	0.956	0.836	1.093	0.5085	0.963	0.774	1.198	0.7341

Severe COVID-19(Yes/No) and Vitamin A								
	Univariate				Multivariate			
Variable	Crude OR	95% CI		p-value	adjusted OR	95% CI		p-value
Age > 75 years	0.595	0.258	1.375	0.2246	0.506	0.129	1.991	0.3299
Male sex	1.510	0.665	3.428	0.3242	0.427	0.109	1.672	0.2219
CRP (mg/L)*	1.005	1.000	1.011	0.0614	0.996	0.985	1.007	0.4841
Vit D supp	0.583	0.245	1.387	0.2225	0.518	0.141	1.893	0.3196
Albumin (g/L) **	0.741	0.646	0.850	**<0.0001**	0.688	0.566	0.836	**0.0002**
BMI ⩾ 35 (kg/m²)	2.765	0.646	11.838	0.1706	15.172	0.951	242.044	0.0543
Vitamin A (µmol/L) #	1.060	0.992	1.132	0.0840	1.022	0.919	1.137	0.4025

Severe COVID-19 (Yes/No) and Beta caroten								
	Univariate				Multivariate			
Variable	Crude OR	95% CI		p-value	adjusted OR	95% CI		p-value
Age > 75 years	0.595	0.258	1.375	0.2246	0.542	0.142	2.075	0.3711
Male sex	1.510	0.665	3.428	0.3242	0.395	0.103	1.515	0.1755
CRP (mg/L)*	1.005	1.000	1.011	0.0614	0.997	0.988	1.007	0.5635
Vit D supp	0.583	0.245	1.387	0.2225	0.620	0.168	2.285	0.4727
Albumin (g/L) **	0.741	0.646	0.850	**<0.0001**	0.706	0.586	0.850	**0.0002**
BMI ⩾ 35 (kg/m²)	2.765	0.646	11.838	0.1706	10.885	0.763	155.310	0.0784
Beta caroten (µmol/L) #	1.421	1.142	1.767	**0.0016**	1.230	0.904	1.672	0.1871

Severe COVID-19 (Yes/No) and Vitamin E								
	Univariate				Multivariate			
Variable	Crude OR	95% CI		p-value	adjusted OR	95% CI		p-value
Age > 75 years	0.595	0.258	1.375	0.2246	0.519	0.130	2.067	0.3522
Male sex	1.510	0.665	3.428	0.3242	0.454	0.117	1.761	0.2536
CRP (mg/L)*	1.005	1.000	1.011	0.0614	0.998	0.989	1.008	0.7254
Vit D supp	0.583	0.245	1.387	0.2225	0.546	0.150	1.983	0.3577
Albumin (g/L) **	0.741	0.646	0.850	**<0.0001**	0.690	0.568	0.838	**0.0002**
BMI ⩾ 35 (kg/m²)	2.765	0.646	11.838	0.1706	12.300	0.894	169.178	0.0606
Vitamin E (µmol/L) ##	1.011	0.965	1.060	0.6393	1.004	0.934	1.079	0.5873

Abbreviations: OR, Odds ratio; BMI, body mass index; Vit D supp, pre-hospital vitamin D supplementation. * for the increase of one mg/L, ** for the increase of one g/L, # for the decrease of 0.1 unit, ## for the decrease of 1 units, ### for the decrease of 10 units. Values of $p < 0.05$ are shown in bold.
